# Soluble B-cell maturation antigen as a monitoring marker for multiple myeloma

**DOI:** 10.3389/pore.2023.1611171

**Published:** 2023-04-28

**Authors:** Ádám Wiedemann, Virág Réka Szita, Róbert Horváth, Attila Szederjesi, Attila Sebő, András Dávid Tóth, Tamás Masszi, Gergely Varga

**Affiliations:** Department of Internal Medicine and Haematology, Semmelweis University, Budapest, Hungary

**Keywords:** multiple myeloma, monitoring, monoclonal gammopathie, soluble B-cell maturation antigen, BCMA

## Abstract

**Objective:** Response to treatment in multiple myeloma (MM) is routinely measured by serum and urine M-protein and free light chain (FLC), as described by the International Myeloma Working Group (IMWG) consensus statement. A non-negligible subgroup of patients however present without measurable biomarkers, others become oligo or non-secretory during recurrent relapses. The aim of our research was to evaluate soluble B-cell maturation antigen (sBCMA) as a monitoring marker measured concurrent with the standard monitoring in MM patients at diagnosis, at relapse and during follow up, in order to establish its potential usefulness in oligo and non-secretory disease.

**Method:** sBCMA levels were measured in 149 patients treated for plasma cell dyscrasia (3 monoclonal gammopathy of unknown significance, 5 smoldering myeloma, 7 plasmacytoma, 8 AL amyloidosis and 126 MM) and 16 control subjects using a commercial ELISA kit. In 43 newly diagnosed patients sBCMA levels were measured at multiple timepoints during treatment, and compared to conventional IMWG response and progression free survival (PFS).

**Results:** sBCMA levels among control subjects were significantly lower than among newly diagnosed or relapsed MM patients [20.8 (14.7–38.7) ng/mL vs. 676 (89.5–1,650) and 264 (20.7–1,603) ng/mL, respectively]. Significant correlations were found between sBCMA and the degree of bone marrow plasma cell infiltration. Out of the 37 newly diagnosed patients who have reached partial response or better per IMWG criteria, 33 (89%) have had at least a 50% drop in sBCMA level by therapy week 4. Cohorts made similarly to IMWG response criteria—achieving a 50% or 90% drop in sBCMA levels compared to level at diagnosis—had statistically significant differences in PFS.

**Conclusion:** Our results confirmed that sBCMA levels are prognostic at important decision points in myeloma, and the percentage of BCMA change is predictive for PFS. This highlights the great potential use of sBCMA in oligo- and non-secretory myeloma.

## Introduction

At first glance, the monitoring of multiple myeloma (MM) seems straightforward compared to other types of cancers. The tumor cells usually keep the innate ability of normal plasma cells (PC) to synthetize large quantities of monoclonal immunoglobulins and/or produce free immunoglobulin light chains (FLC), which are present in the serum of patients and can then be utilized to define the well-established International Myeloma Working Group (IMWG) response criteria (1, 2). However, approximately 3% of MM patients present without conventionally measurable serum markers. Several different etiologies can account for this occurrence. Defects in the immunoglobulin synthesis result in non-producer patients, in whom even the use of free light chain assay will not reveal measurable disease (3–7). Another subgroup of patients has defects in the secretion of immunoglobulins, leading to oligosecretory multiple myeloma. The defining criteria for these patients (serum M-protein of <10.0 g/L, urine M-protein of <200 mg/24 h, and free light chain of <100 mg/L) can make reliable measurements and therefore the monitoring of disease activity challenging. This is a particularly common problem in AL amyloidosis. The exact frequency of non- and oligosecretory myeloma is difficult to quantify, as with each subsequent relapse the tendency of clonal evolution into FLC only or true non-secretory myeloma increases. This often goes parallel with the development of other high risk features, such as genomic instability, extramedullary disease manifestations and drug resistance (8).

Oligo- and non-secretory myeloma patients present a problematic subgroup. Their diagnosis is frequently delayed; they do not meet the inclusion criteria of most clinical trials. To objectively measure response to treatment, FDG PET-CT and bone marrow biopsy are the only techniques currently being used, making the evaluation of the optimal timing to end or restart active therapy a challenge. The nature of these modalities also prevent them from being repeated on any regular basis.

B-cell maturation antigen (BCMA) is a member of the tumor necrosis factor receptor superfamily, present almost exclusively on the surface of mature B-cells and plasma cells. Recently it became a popular target for the development of cellular and non-cellular immunotherapies in MM, several of which are now licensed for treatment, such as belantamab mafodotin (9), ciltacabtagene autoleucel (10), idecabtagene vicleucel (11), and teclistamab (12).

BCMA is actively cleaved from the plasma cell surface by the ubiquitous multisubunit gamma-secretase complex, which then releases a soluble BCMA fragment (sBCMA) into the serum (13). Soluble BCMA was evaluated at first from the viewpoint of BCMA-targeted therapies: cleavage not only leads to the reduction of ligand density on tumor cells, sBCMA is capable of inhibiting CAR-T-cell function as well. Decreasing sBCMA levels can be a sign of biallelic loss of the BCMA target (14), whereas the reappearance of sBCMA can indicate the elimination of the anti-BCMA CAR-T-cell clone (15). The use of sBCMA as a prognostic and/or monitoring tool was suggested in non-BCMA targeted therapies as well. The levels of sBCMA not only positively correlate with disease stage, its increase was also reported through the progression from monoclonal gammopathy of unknown significance (MGUS) to active MM (16, 17). In another study, sBCMA levels were shown to correlate with the proportion of plasma cells found in bone marrow biopsies, the quality of response to therapy, and were prognostic towards progression free survival (PFS) and overall survival (OS) as well (18). Importantly, serum B-cell maturation antigen levels do not show any dependence on renal function, like free light chains do. Compared to M-protein, sBCMA has a drastically shorter plasma half-life (24–36 h), permitting rapid changes in measurable serum values, which could in turn reflect the ongoing changes in plasma cell burden, making it a potentially great monitoring tool (18).

The aim of our research was to evaluate sBCMA as a monitoring marker measured concurrent with the standard monitoring in MM patients at diagnosis, at relapse and during follow up, in order to establish its potential usefulness in oligo and non-secretory disease.

## Materials and methods

### Patients

Peripheral blood samples were obtained from patients with MM at their standard clinic visits parallel with the conventional monitoring strategy, and were kept frozen at −95°C until evaluation. Standard monitoring included routine full blood count and chemistry, serum and urine electrophoresis (SPEP and UPEP), serum FLC, and in addition immunofixation (IFX) when needed. These assays were performed in the hospital’s routine laboratory and served as benchmark values for the study. Newly diagnosed and relapsed patients had follow up tests 1 week after treatment initiation (±3 days) and then monthly (±7 days).

Samples were collected after written consent from 3 MGUS, 5 smoldering MM, 7 plasmacytoma, 8 AL amyloidosis and 126 MM patients, as well as 16 normal controls, who were included in our research (Table 1.). Out of the 126 MM patients, 90 had multiple measurements evaluated. 43 out of these were newly diagnosed, being treated with bortezomib-thalidomide-dexamethasone (VTD) protocol, which was the standard approach in Hungary at the time.

**TABLE 1:** Subjects included in the analysis.

Category	Number of subjects
Healthy control	16
MGUS	3
Smouldering myeloma	5
Amyloidosis	8
Plasmacytoma	7
MM	126
Newly diagnosed	56
Relapsed	19
Follow up	51

### ELISA assay

Frozen serum samples were thawed and diluted 1:500. An enzyme-linked immunosorbent assay with a polyclonal anti-BCMA antibody (Human BCMA/TNFRSF17 DuoSet ELISA DY193—R&D Systems, Minneapolis, MN, United States) was used to measure the sBCMA levels, as previously published (19). The ELISA plates were read by IL Biokit ELx800 ELISA Reader (Werfen Company, Barcelona, Spain) plate reader at 450 nm using Biotek Gen5 1.11 software.

## Response categories and statistics

Soluble BCMA levels were then analyzed both as absolute values and also as percentage of the original reading to indicate response. We followed the way IMWG defined response categories based on the change from initial M-protein and FLC, i.e., as 50% and 90% drop from the original level to define sBCMA response categories sBCMA-SD, PR and VGPR.

The overall response rate was defined as the collective proportion of patients with complete response (CR), very good partial remission (VGPR), partial remission (PR), or stable disease (SD) as their best response (1). The primary endpoint for outcome was PFS which was measured from start of treatment to disease progression or death. Comparisons of dichotomous variables were performed by Fisher’s exact test. Continuous variables were compared using Mann-Whitney U test and Spearmann correlation analysis. PFS and OS were estimated by Kaplan–Meier analysis. The analyses were carried out using the SPSS (version 20.0; SPSS, Chicago, IL) software package.

The study was approved by the Institutional Review Board and informed consent was obtained in accordance with the Declaration of Helsinki.

## Results

### Soluble BCMA levels correlated with disease stage

**FIGURE 1:**
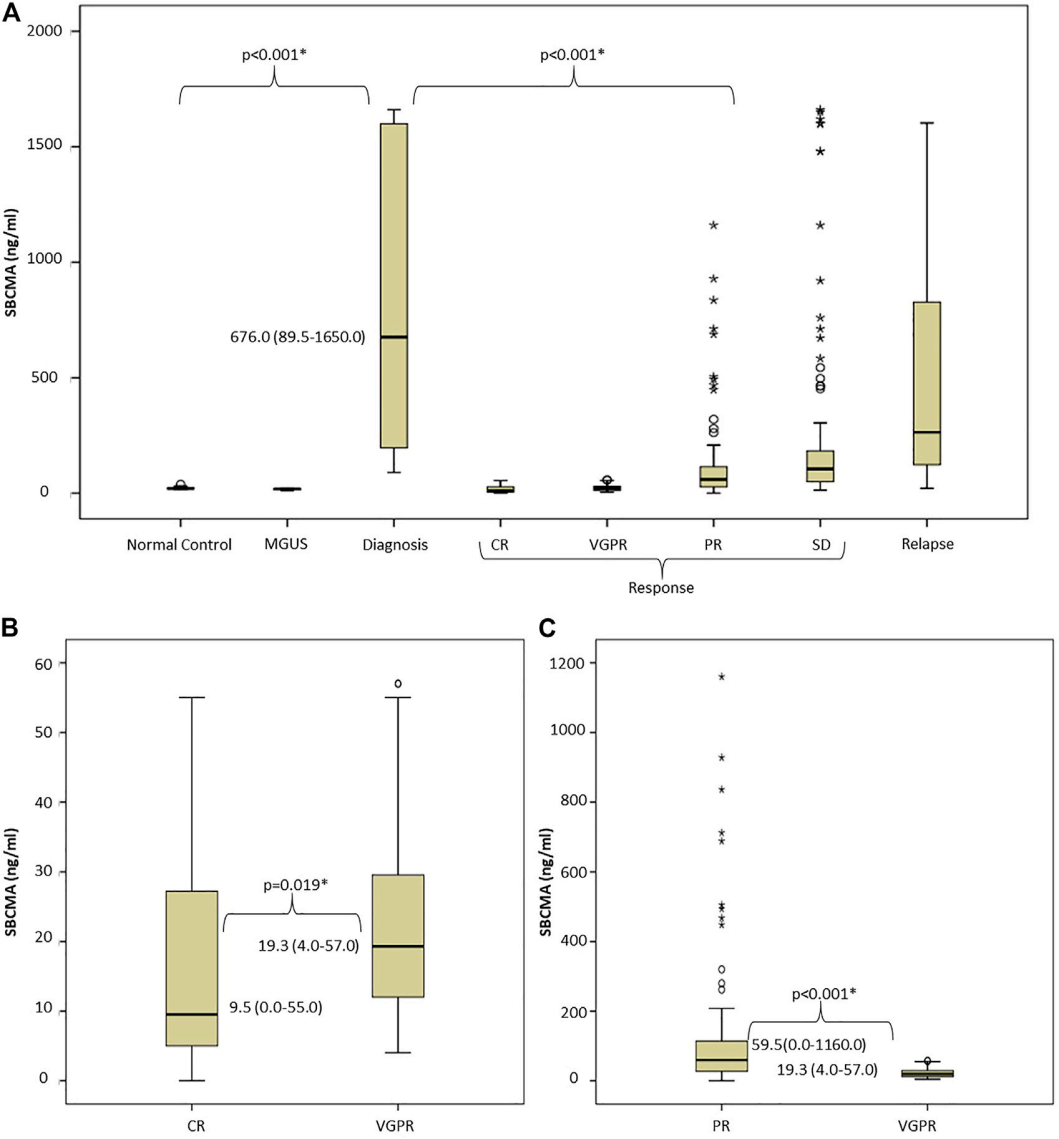
Evaluation of soluble BCMA levels: **(A)** There was significant difference between normal controls, MGUS cases, and MM patients with active disease or in remission, **(B,C)** Paired analysis of sBCMA levels using Mann-Whitney U test shows significant difference between IMWG response groups Abbreviations: sBCMA, soluble B-cell maturation antigen; MGUS, monoclonal gammopathy of undetermined significance; DG, diagnosis; CR, complete remission; VGPR, very good partial response; PR, partial response; SD, stable disease; PD, progressive disease; IMWG, International Myeloma Working Group.

The median sBCMA level among the normal controls was 20.8 (14.7–38.7) ng/mL, in the MGUS cases 18.1 (12.0–20.5) ng/mL. In newly diagnosed, as well as relapsed patients the sBCMA levels were significantly higher: median 676.0 (89.5–1650.0) and 264 (20.7–1603.0) ng/mL respectively. In responding patients, the median sBCMA levels were significantly lower: patients in CR had 9.5 (0.0–55.0) ng/mL, VGPR 19.3 (4.0–57.0) ng/mL, PR 59.5 (0.0–1160.0) ng/mL, SD 104.5 (13.5–1660.0) ng/mL. There were more outliers in the more broadly defined IMWG response categories (i.e., PR and SD). Importantly, in paired analysis using Mann-Whitney U test, there were statistically significant differences between all categories of active disease and response, as well as between normal or MGUS and active myeloma (Figures 1A–C).

We found significant correlations between sBCMA level and bone marrow PC infiltration (BM biopsy PC% and sBCMA Spearman *r* = 0.6376; *p* = 0.0044, BM aspiration PC% and sBCMA Spearman *r* = 0.5203; *p* = 0.0054) and sBCMA and B2MG levels (Spearman *r* = 0.3643; *p* =0.01). Lactate dehydrogenase levels were unfortunately not collected systematically in all cases, so we did not have the power to analyze its connection with sBCMA.

### Changes in BCMA levels during treatment in responding patients

**FIGURE 2:**
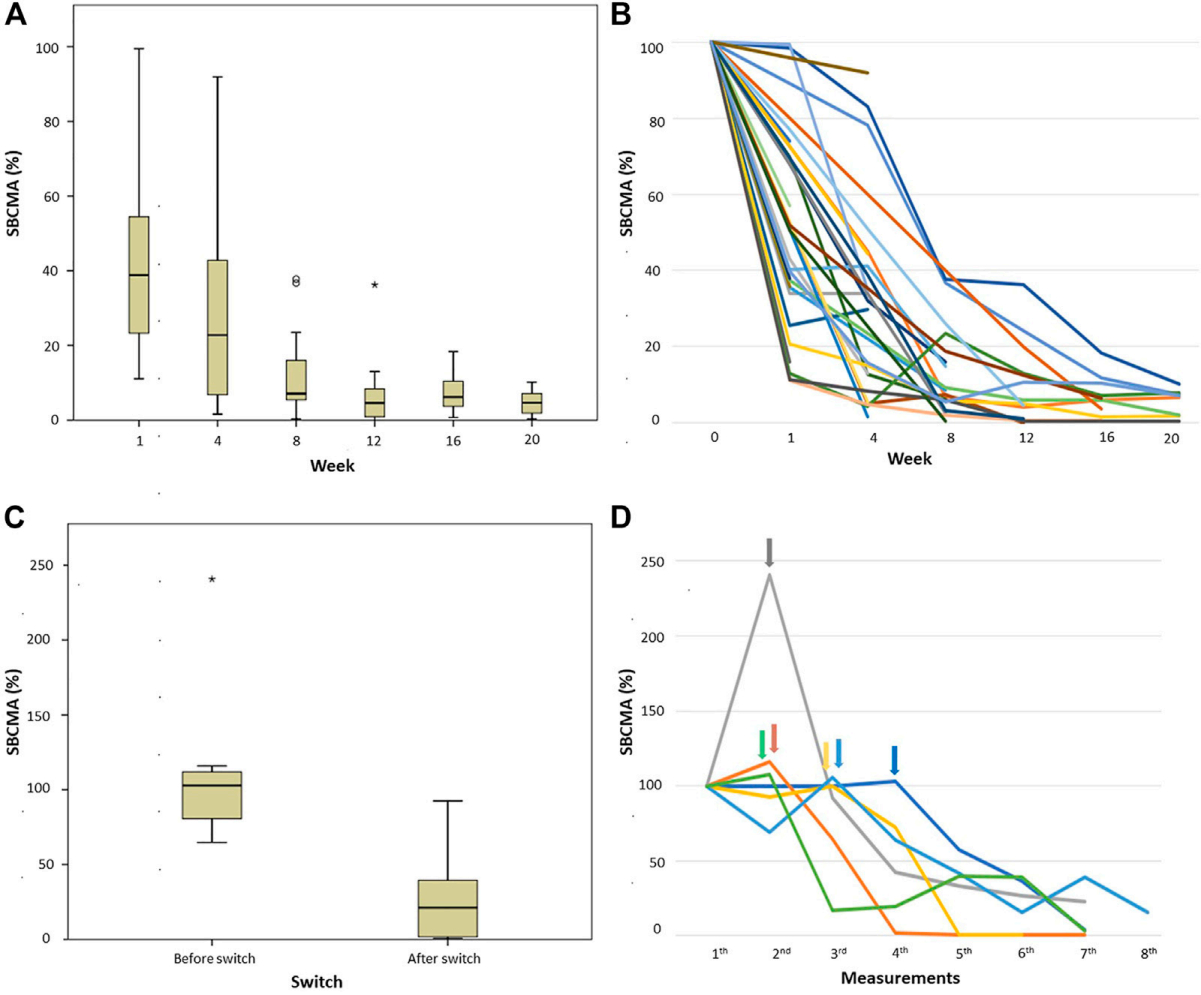
sBCMA levels measured in patients who had induction treatment with VTD protocol. **(A,B)** percentage of sBCMA drop in patient achieved IMWG-PR or better response compared to day 0 (100%), **(A)** box and whisker plot and **(B)** graphic representation of individual cases; and **(C,D)** patients not achieving PR after first line treatment and therefor having salvage treatment, **(C)** box and whisker plot of percentage of sBCMA change before and after switch to salvage treatment compared to presentation (100%), **(D)** graphic representation of the individual cases, arrows indicating the time of treatment change. Abbreviations: sBCMA, soluble B-cell maturation antigen; PR, partial response.

37 newly diagnosed patients were treated with VTD protocol and reached at least partial response according to IMWG criteria, as well as had BCMA levels measured at least twice following treatment initiation. At week one, 30 of these patients had a sBCMA measurement, 19 of whom had an at least 50% drop by this time compared to the initial result. All 37 patients had a repeat BCMA level measurement by week 4 (done either on week 1 or week 4), and only 4 of them failed to show more than 50% drop. For a graphic illustration of these responses see Figures 2A, B. The difference between week 0 measurements and any other timepoints was statistically significant.

**FIGURE 3:**
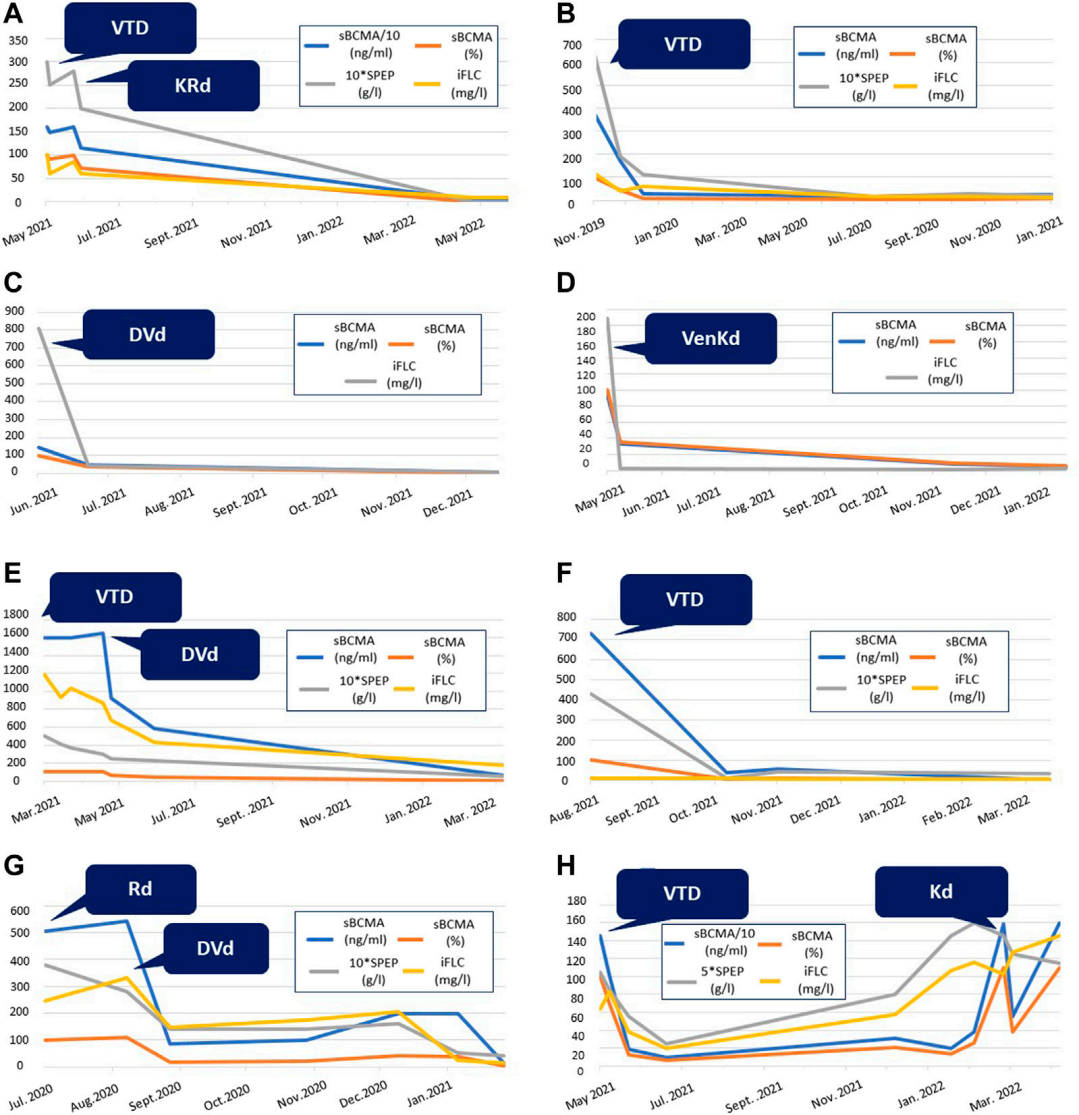
Graphic comparison of the changes in conventional myeloma biomarkers and sBCMA levels during the treatment of eight different patients from our cohort. SPEP is multiplied by 10 in selected diagrams for easier representation. Abbreviations: sBCMA, soluble B-cell maturation antigen; SPEP, serum protein electrophoresis; iFLC, involved free light chain; VTD, bortezomib-thalidomide-dexamethason; VenKD, venetoclax-carfilzomib-dexamethason; KRd, carfilzomib-lenalidomid-dexamethason; DVd, daratumumab-bortezomib-dexamethason; KD, carfilzomib-dexamethason; Rd, lenalidomid-dexamethason.

sBCMA levels correlated with changes in M-protein and serum FLC levels measured at the same timepoints in individual patients. See Figures 3A–G for eight examples.

### Changes in BCMA levels in patients requiring salvage treatment and in relapse

There were 6 newly diagnosed patients who failed to reach PR or progressed on VTD treatment, requiring a change to salvage therapy (to daratumumab-bortezomib-dexamethasone in 3, bortezomib-lenalidomide-dexamethasone in 1, carfilzomib-lenalidomide-dexamethasone in 1 and carfilzomib-venetoclax-dexamethasone in 1 case). Figures 2C, D shows sBCMA levels before and after these treatment changes demonstrating that all of them reached at least sBCMA-PR after switch.

During the follow up, 6 further patients relapsed biochemically based on M-protein and/or sFLC measurements, according to IMWG criteria. Parallel with this, each of these cases showed a marked rise in their sBCMA, with at minimum a doubling of their initial value.

### Month 1 sBCMA level changes define cohorts of patients with distinct survival probabilities

**FIGURE 4:**
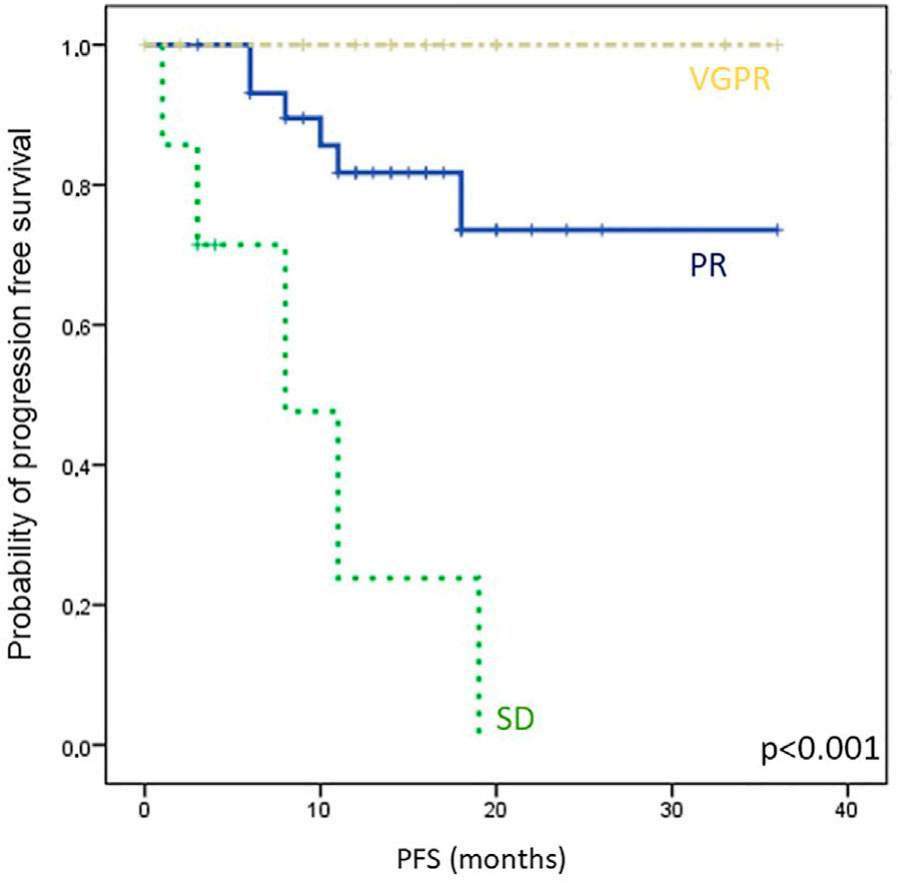
Kaplan-Meier analysis of progression free survival (PFS) in different sBCMA response groups, classified by sBCMA levels after 4 weeks of treatment. Abbreviations: sBCMA, soluble B-cell maturation antigen; VGPR, very good partial response; PR, partial response; SD, stable disease.

All patients who had week 0 and week 4 (±7 days) sBCMA measurements were included in this part of the analysis. We followed the IMWG standard for the analysis of M-protein and sFLC changes, and used the 50% and 10% sBCMA change thresholds to define response categories of sBCMA-SD, sBCMA-PR and sBCMA-VGPR. Following this, we analyzed PFS in these subgroups, and found marked differences (PFS not reached in responding patients and only 5 months in those not reaching sBCMA-PR, *p* < 0.001, Figure 4).

### Non-secretory patients, localized relapse and amyloidosis

Only two of the follow up patients were true non-secretory, and one had oligosecretory disease, none of them showed signs of relapse. We had limited experience with only plasmocytic relapse which was observed in 6 patients. All 6 had at least doubling of the sBCMA level, compared to nadir exceeding the 95% confidence interval of CR and VGPR patients, but the absolute rise was small (<100 ng/mL). The number of patients with amyloidosis was also two small to draw any conclusions, but BCMA levels were lower in this group (<100 ng/mL in all but one case).

## Discussion

Our results indicate the feasibility of using sBCMA for the monitoring of MM in conjunction with or—in the case of nonsecretory MM—instead of standard SPEP, UPEP and FLC measurements.

We successfully used the conventional 50% and 10% thresholds when segregating sBCMA response into SD, PR and VGPR groups, and found at least 100% rise from baseline in all cases of relapse. We think that sBCMA used this way could be a valuable addition to standard monitoring, especially in non- and oligosecretory disease. The problem with cases relapsing as oligo- or non-secretory is that lacking early biochemical warning signs, we are forced to wait until patients become outright symptomatic—often suffering serious consequences, such as skeletal fractures—until we may know they are truly relapsing. This is in part because mild variations in the M-protein and FLC levels are not uncommon, and moderate relapse symptoms such as anaemia, renal failure and bone pain may be related to other diseases or pre-existing conditions. In such situations, another layer of information could prove invaluable, giving a warning signal to prompt more frequent monitoring, bone marrow analysis or PET-CT.

Mass spectrometry was recently used in some of the leading laboratories to replace gel electrophoretic techniques (20, 21). Matrix-assisted laser desorption/ionization time of flight (MALDI TOF) mass spectrometry is several magnitudes more sensitive compared to the standard monitoring (i.e., SPEP, IFX, UPEP and FLC used together), reaching sensitivity similar to bone marrow based MRD techniques (22). Still, these promising techniques are only available in a handful of outstanding laboratories, and this situation is not expected to change in the near future. Possible limitations are the large initial cost of the installation of a mass spectrometer, and the lack of sufficient expertise in most laboratories. In addition to these, the current IMWG categories are established using gel techniques, and replacing them with mass spectrometry based measurements would require standardization as well as large scale validation.

Another potential candidate to improve myeloma monitoring is the Hevylite assay, developed by Binding Site, the same company that produced Freelite. For obvious reasons Hevylite will not be of help with light chain only oligosecretory disease, but can increase the sensitivity of monoclonal IgA and IgG detection at the point when SPEP and IFX cannot give a numerical value any longer due to the presence of polyclonal immunoglobulins (23). As a result Hevylite was included in the IMWG monitoring guideline in 2016, but so far did not get accepted generally in the routine clinical practice (24).

One important issue with FLC as a monitoring tool is that what we actually measure is the total FLC level and not the monoclonal fraction, and both kappa and lambda FLC levels are affected by renal clearance as well as immune activation. As renal failure is one of the important hallmarks of multiple myeloma, the abnormally elevated polyclonal FLC background very frequently makes FLC interpretation difficult, if not impossible. As Ghermezi et al. demonstrated, sBCMA levels are not dependent on renal function, and therefore can eliminate this important complication.

Another protein expressed on the surface of plasmacells similarly to BCMA is CD-138 (Syndecan) which is also shed into serum. This marker was suggested as independent prognostic parameter in multiple myeloma (25, 26), and the drop of serum level correlated with outcome in a cohort of patients (26, 27).

We did not include oligo and non-secretory patients in this analysis, and the benchmark we compared sBCMA response to, were the standard biomarkers IMWG suggests to use. We believe that our findings can still be generalized and utilized in oligo and non-secretory MM. Further studies enrolling oligo and non-secretory MM patients are warranted.

In conclusion, sBCMA is a cheap, noninvasive and easy to utilize ELISA measurement to monitor myeloma suggested to be used when the current monitoring strategy cannot give a reliable result.

## Data Availability

The raw data supporting the conclusion of this article will be made available by the authors, without undue reservation.
